# Nursing intervention and follow-up compliance after abnormal cervical cancer screening

**DOI:** 10.1097/MD.0000000000049893

**Published:** 2026-08-21

**Authors:** Xiaohui Deng, Xiaoqing Pang, Zhengyan Dai

**Affiliations:** a Department of Gynecology and Obstetrics, Shijiazhuang Maternity and Child-Health Hospital, Shijiazhuang, Hebei, China.

**Keywords:** abnormal screening results, cervical cancer, follow-up compliance, nursing intervention, retrospective time-grouped cohort study

## Abstract

This study aimed to explore the impact of systematic nursing intervention on follow-up compliance among patients with abnormal cervical cancer screening results. This was a single-center, retrospective, time-grouped cohort study. A total of 182 patients who received cervical cancer screening at our hospital from June 2023 to June 2025 and had abnormal results requiring further follow-up were enrolled. According to the implementation timing of the nursing program, patients from June 2023 to May 2024 were included in the control group (routine nursing, 91 cases), and patients from June 2024 to June 2025 were included in the intervention group (systematic nursing intervention, 91 cases). The primary outcome was short-term follow-up compliance, defined as completion of colposcopy or treatment within 3 months after notification. Follow-up compliance rates, adherence levels, and reasons for loss to follow-up were compared. Univariate and multivariate logistic regression analyses were performed to explore influencing factors, and potential time-period bias was considered when interpreting the results. The follow-up compliance rate in the intervention group was 86.81%, significantly higher than that in the control group (64.84%) (*P* < .001). Regarding reasons for loss to follow-up, the control group was primarily due to forgetting (34.38%) and fear/anxiety (25.00%), while the intervention group was mainly due to being busy with work (33.33%) and inconvenient transportation (25.00%) (*P* = .026). Multivariate analysis showed that nursing intervention was independently associated with improved follow-up compliance (odds ratio [OR] = 4.128, *P* < .001); age ≥51 years (OR = 0.312, *P* = .010), education level of junior college or above (OR = 3.245, *P* = .014), and urban residence (OR = 2.436, *P* = .020) were also independent influencing factors. Subgroup analysis indicated that nursing intervention improved compliance rates across all populations, with more significant effects observed in human papillomavirus-positive and rural populations. Systematic nursing intervention was associated with improved short-term follow-up compliance among patients with abnormal cervical cancer screening results. Because this study used a retrospective cohort based on time grouping and was conducted in a single medical institution, the findings should be interpreted cautiously and applied primarily to similar clinical settings.

## 1. Introduction

Cervical cancer is one of the common malignant tumors among women globally, posing a serious threat to women’s health. According to statistics, there were approximately 604,000 new cases of cervical cancer and about 342,000 deaths worldwide in 2020, the majority of which occurred in developing countries.^[1]^ The incidence and mortality rates of cervical cancer in China are both on the rise, showing a trend of affecting younger populations, and it has become a significant public health issue.^[2]^

Cervical cancer is one of the few malignant tumors with a clear etiology that can be effectively prevented and controlled through early screening and intervention. Persistent infection with human papillomavirus (HPV) is the main causative factor for cervical cancer. It typically takes 5 to 10 years for precancerous lesions to develop into invasive cancer, which provides a critical time window for early diagnosis and treatment.^[3]^ Numerous studies have shown that standardized cervical cancer screening and timely follow-up intervention for patients with abnormal screening results can significantly reduce the incidence and mortality of cervical cancer.^[4,5]^

However, in the efforts towards early diagnosis and treatment of cervical cancer, poor follow-up compliance among patients with abnormal screening results is a major bottleneck restricting the effectiveness of prevention and control. Studies from both domestic and international sources show that the rate of loss to follow-up for patients with abnormal screening results ranges from 30% to 50%.^[6,7]^ Failure to complete colposcopy or treatment on time may lead to the progression of precancerous lesions to invasive cancer, resulting in the loss of the optimal treatment window. Analysis of the reasons reveals multiple factors, including patients’ insufficient understanding of screening results, fear of subsequent examinations, forgetting scheduled appointments, being busy with work, inconvenient transportation, and the belief that follow-up is unnecessary due to the absence of symptoms.^[8,9]^

As an extension of medical services, nursing intervention plays a significant role in improving patient treatment compliance. In recent years, scholars domestically and internationally have explored various nursing intervention measures, including establishing follow-up records, intensifying health education, telephone and SMS reminders, and psychological counseling. Preliminary studies indicate that these measures can help improve patient follow-up compliance.^[10]^ However, current related research mostly focuses on areas such as breast cancer, hypertension, and diabetes. Systematic studies on nursing interventions specifically for patients with abnormal cervical cancer screening results are relatively scarce, and there is a lack of evidence-based proof derived from retrospective cohort study designs.^[11]^

Based on this, the present study used a single-center retrospective cohort design based on time grouping. Patients with abnormal cervical cancer screening results requiring further follow-up at our hospital were enrolled as the study subjects. According to the timing of implementation of systematic nursing intervention, patients treated before full implementation were assigned to the control group and those treated after implementation were assigned to the intervention group. The follow-up compliance rates and reasons for loss to follow-up were compared, factors influencing follow-up compliance were analyzed, and the differential effects of nursing intervention across various populations were evaluated. Because grouping was determined by calendar period rather than random allocation, this study was designed to evaluate the association between implementation of a nursing intervention package and follow-up compliance, rather than to establish definitive causality. The aim was to provide evidence-based support for optimizing nursing workflows in early diagnosis and treatment of cervical cancer and improving the quality of follow-up management.

## 2. Methods

### 2.1. Study subjects

This study was approved by the Ethics Committee of Shijiazhuang Maternal and Child Health Hospital. A retrospective cohort study based on time grouping was adopted. Patients who underwent cervical cancer screening at our hospital from June 2023 to June 2025 and had abnormal results requiring further follow-up were selected as the study subjects. According to the implementation timeline of nursing intervention measures at our hospital, the study subjects were divided into 2 groups: the control group (pre-implementation): patients admitted from June 2023 to May 2024 before the full implementation of nursing intervention measures, who received routine medical advice and follow-up notifications; and the intervention group (post-implementation): patients admitted from June 2024 to June 2025 after the full implementation of nursing intervention measures, who received systematic nursing intervention in addition to routine diagnosis and treatment.

Because the control and intervention groups came from sequential calendar periods, calendar-time-related differences may have influenced the results. Potential sources of time bias include changes in patient awareness of HPV and cervical cancer screening, clinic workload, appointment availability, staffing experience, or institutional follow-up procedures over time. To reduce this potential bias, both groups were derived from the same hospital, the definitions of abnormal screening results and eligibility criteria were kept consistent, baseline demographic and clinical characteristics were compared, and multivariate models adjusted for available covariates. Nevertheless, residual temporal confounding could not be completely excluded, and the findings were interpreted cautiously in the Discussion.

### 2.2. Inclusion and exclusion criteria

Inclusion criteria: records indicated undergoing cervical cancer screening at our hospital between June 2023 and June 2025; laboratory/pathology reports showed initial screening results meeting any of the following abnormal criteria, requiring subsequent colposcopy, pathological biopsy, or treatment according to the clinical guidelines at the time: positive high-risk HPV test (including HPV 16/18 positive or other high-risk subtypes positive); cervical thinprep cytologic test (TCT) report showing atypical squamous cells of undetermined significance or higher lesions; combined HPV genotyping and TCT results indicated a need for further diagnostic clarification; age recorded was between 18 and 65 years; complete key information, including basic patient information, contact details, and screening results, was recorded in the medical record system.

Exclusion criteria: previous medical records indicated a confirmed diagnosis of cervical cancer or cervical intraepithelial neoplasia grade II or higher, and related surgical treatment had been received; records indicated the patient was pregnant or in the postpartum period at the time of screening; key results were missing from the screening report, or no valid contact information was recorded in the file, making it impossible to trace the follow-up outcome; medical or follow-up records explicitly stated that the patient was transferred to another hospital for subsequent diagnosis and treatment, precluding inclusion in our hospital’s follow-up statistics.

### 2.3. Sample size calculation

To ensure the statistical power of the retrospective data analysis, a sample size estimation was performed prior to data collection in this study. Using the follow-up compliance rate as the primary outcome indicator, and based on a review of previous literature and preliminary follow-up data statistics from our hospital, the follow-up compliance rate was estimated to be approximately 60% for the control group (routine nursing period) and approximately 80% for the intervention group (nursing intervention period). With a set significance level of α = 0.05 (two-tailed) and a test power of 1-β = 0.80, the sample size calculation formula for comparing 2 independent sample proportions was applied, yielding a requirement of approximately 79 cases per group. Considering the possibility of missing data or incomplete records in a retrospective study, the calculated value was increased by 15%, ultimately determining the inclusion of 91 cases per group.

### 2.4. Nursing grouping assessment

In this study, enrolled subjects were divided into a control group (pre-intervention) and an intervention group (post-intervention) based on the nursing implementation phase during which they attended the hospital. The specific nursing measures received by patients in the 2 groups are as follows.

### 2.5. Nursing methods for the control group

The control group consisted of patients admitted from June 2023 to May 2024, who received routine nursing care after cervical cancer screening. After the screening results were issued, the outpatient doctor verbally informed the patient of the results during a follow-up visit or via telephone notification, briefly explaining the necessity for further colposcopy or treatment. Concurrently, patients were provided with standardized cervical cancer prevention and treatment informational brochures, covering general scientific knowledge on the etiology of cervical cancer and the significance of screening. One-on-one intensive education specifically for patients with abnormal results was not implemented. Follow-up was conducted passively; doctors or nurses made routine inquiries during outpatient work hours, and an active follow-up reminder mechanism was not established. Patients were required to return for follow-up visits at the time recommended by the doctor on their own, and the hospital only provided answers when patients proactively called for consultation. Psychological support was limited to simple verbal reassurance for patients who exhibited significant anxiety during their visit, with no standardized psychological assessment or intervention process.

### 2.6. Nursing methods for the intervention group

The intervention group consisted of patients admitted from June 2024 to June 2025. In addition to routine nursing care, they received a systematic nursing intervention package implemented by trained specialized nurses. On the day the abnormal screening result was reported, a specialized nurse established an electronic follow-up record for the patient, meticulously documenting basic information, contact details, screening results, scheduled follow-up appointment time, etc, and flagged key subjects for focused attention. Regarding health education, using face-to-face or telephone communication, the significance of the screening result was explained to the patient in plain language, with emphasis on the concepts that “ precancerous lesion ≠ cancer” and the curability associated with “ early diagnosis and treatment,” aiming to correct the patient’s fears. Concurrently, a self-developed “ Cervical Precancerous Lesion Follow-up Guidance Manual” was provided, containing a schematic diagram of the colposcopy procedure, precautions, and a reminder card for follow-up appointments. In terms of follow-up management, one week prior to the scheduled follow-up appointment date, a reminder notification was sent by the specialized nurse via telephone or SMS platform, informing the patient of the follow-up time, location, and precautions. For patients who did not attend the appointment within one week of the scheduled time, the nurse made another telephone contact to understand the reason for nonattendance and reschedule the appointment. For those who could not be contacted after multiple attempts or explicitly refused follow-up, the reason for loss to follow-up was documented. Regarding psychological support, during the initial communication, the patient’s psychological state was assessed using a self-rating anxiety scale or through an interview, and psychological counseling was provided using techniques such as listening, empathy, and encouragement to enhance the patient’s confidence. Furthermore, patients in the intervention group were offered priority scheduling for colposcopy or treatment, aiming to minimize waiting times and reduce dropout rates associated with cumbersome consultation procedures.

### 2.7. Comparison of differences in nursing methods between the 2 groups

The main differences in nursing methods between the 2 groups are reflected in the following aspects: the control group primarily relied on passive notification and patients returning for follow-up on their own, whereas the intervention group established active follow-up records and implemented mechanisms for advance reminders and follow-up for overdue appointments; the control group only distributed general informational brochures, while the intervention group employed one-on-one explanations combined with illustrated manuals for intensified health education; the control group provided only simple reassurance when patients exhibited anxiety, whereas the intervention group conducted standardized psychological assessments and provided continuous support throughout the process; additionally, the intervention group offered patients priority access via a green channel for appointment scheduling, while the control group followed routine procedures for visits.

## 3. Data collection

### 3.1. Collection of general demographic data

General demographic data of the patients were collected through the Hospital Information System (HIS) and cervical cancer screening records. This mainly included age, educational level, marital status, place of residence, occupation, annual family income, and type of health insurance. Age was determined based on the patient’s ID card information and categorized into 3 groups as required by the study: ≤35 years, 36 to 50 years, and ≥51 years. Educational level, based on the highest degree recorded at the time of the visit, was classified into 3 categories: junior middle school or below, high school/technical secondary school, and junior college or above. Marital status was divided into 3 groups: married, unmarried, and divorced/widowed. Place of residence was classified as urban or rural based on registered residence or current address. Occupational status was categorized as employed or unemployed/retired. Annual family income, based on the income level reported by the patient or their family, was divided into 3 tiers: <50,000 yuan, 50,000 to 100,000 yuan, and >100,000 yuan. Type of health insurance was classified into 3 categories based on the insurance used by the patient during the visit: employee health insurance, resident health insurance, and self-pay.

### 3.2. Collection of clinical characteristic data

Cervical cancer screening results and related clinical data of the patients were collected through the laboratory and pathology information systems. Screening results mainly included high-risk HPV test results and Cervical TCT results, classified into 3 categories based on report records: HPV-positive, TCT abnormal, and double positive. Concurrently, patients’ previous screening history (whether they had undergone cervical cancer screening before), family history of cervical cancer (whether a first-degree relative had a history of cervical cancer), and comorbidities were collected through medical history records. Comorbidities, including hypertension, diabetes, other gynecological diseases (such as uterine fibroids, ovarian cysts, vaginitis, etc), and immune system diseases (such as rheumatoid arthritis, systemic lupus erythematosus, etc), were extracted based on diagnoses documented in the medical records.

### 3.3. Collection of follow-up data

Patients’ follow-up completion status and reasons for loss to follow-up were collected through cervical cancer follow-up management records and outpatient visit records. Follow-up compliance was determined based on whether the patient completed colposcopy or treatment within the specified time window, defined in this study as within 3 months after receiving the notification, and was recorded as compliant or noncompliant. This indicator represented short-term compliance after abnormal screening results. Long-term compliance outcomes, such as 6-month or 12-month sustained follow-up, repeat surveillance, or treatment completion beyond the initial diagnostic window, were not available in the retrospective records and were therefore not included in the outcome analysis. Based on the time of follow-up completion, compliance was further categorized into 3 levels: complete compliance (completed on time), partial compliance (delayed but eventually completed), and noncompliance (not completed). For patients who did not complete follow-up, reasons for loss to follow-up were traced through records in the follow-up registration form or telephone re-inquiry, mainly including forgetting, fear/anxiety, being busy with work, inconvenient transportation, believing follow-up unnecessary due to absence of symptoms, and other reasons.

## 4. Bias control and data quality assessment

To reduce selection bias, all consecutive eligible patients during the defined pre-intervention and post-intervention periods were screened according to prespecified inclusion and exclusion criteria, and cases with missing key variables or invalid contact information were excluded before analysis. To reduce information bias, demographic and clinical variables were extracted from the HIS, laboratory information system, pathology information system, and follow-up registration system using uniform definitions; ambiguous records were checked by 2 trained investigators. Follow-up compliance was based on documented completion of colposcopy or treatment within the 3-month window rather than on patient self-report. The possibility of residual selection bias, information bias, and recall bias was considered when interpreting the findings.

### 4.1. Statistical analysis

SPSS 26.0 statistical software was used for data analysis. Count data were expressed as frequencies and percentages (n [%]), and comparisons between groups were performed using the chi-square test. Measurement data were expressed as mean ± standard deviation (x̄ ± s), and comparisons between 2 groups were performed using the independent samples *t* test. In univariate analysis, with follow-up completion status as the dependent variable, the chi-square test was used to screen for factors potentially influencing follow-up compliance. Variables with *P* < .10 in the univariate analysis were included in a multivariate logistic regression model to analyze independent factors associated with follow-up compliance, and odds ratios (OR) with their 95% confidence intervals were calculated. Because grouping was aligned with calendar-time, intervention exposure was interpreted together with baseline balance and adjusted regression results; however, the design did not allow full separation of intervention effects from secular changes. Further subgroup analyses were conducted according to age, screening results, and place of residence, using the chi-square test to compare differences in follow-up compliance rates between the 2 groups within different subgroups. All tests were two-tailed, and a *P*-value < .05 was considered statistically significant.

## 5. Results

### 5.1. Comparison of baseline characteristics between the 2 groups at enrollment

A total of 182 patients were included in this study, with 91 cases in the control group and 91 cases in the intervention group. Comparisons between the 2 groups in terms of age (42.38 ± 9.64 years vs 43.15 ± 10.02 years), educational level, marital status, place of residence, occupation, annual family income, and type of health insurance showed no statistically significant differences (*P* > .05) (Table 1). Regarding clinical characteristics, the distribution of screening results (HPV-positive, TCT abnormal, double positive), previous screening history, and family history of cervical cancer also showed no significant differences between the 2 groups (*P* > .05). Concerning comorbidities, the prevalence rates of hypertension, diabetes, other gynecological diseases, and immune system diseases were balanced and comparable between the 2 groups (*P* > .05).

**Table 1:** Comparison of general demographic characteristics and clinical baseline data between the 2 groups (n[%]).

Item	Control group (n = 91)	Intervention group (n = 91)	*χ*^2^/*t* value	*P*-value
Age (yr, x̄ ± s)	42.38 ± 9.64	43.15 ± 10.02	0.527	.599
Educational level			1.284	.526
Junior middle school or below	38 (41.76)	35 (38.46)		
High school/technical secondary school	32 (35.16)	30 (32.97)		
Junior college or above	21 (23.08)	26 (28.57)		
Marital status			0.562	.755
Married	76 (83.52)	78 (85.71)		
Unmarried	8 (8.79)	6 (6.59)		
Divorced/widowed	7 (7.69)	7 (7.69)		
Place of residence			0.634	.426
Urban	52 (57.14)	56 (61.54)		
Rural	39 (42.86)	35 (38.46)		
Occupation			0.198	.656
Employed	58 (63.74)	60 (65.93)		
Unemployed/retired	33 (36.26)	31 (34.07)		
Annual family income			0.879	.644
<50,000 yuan	34 (37.36)	30 (32.97)		
50,000–100,000 yuan	41 (45.05)	42 (46.15)		
>100,000 yuan	16 (17.58)	19 (20.88)		
Type of health insurance			1.047	.592
Employee health insurance	47 (51.65)	51 (56.04)		
Resident health insurance	36 (39.56)	34 (37.36)		
Self-pay	8 (8.79)	6 (6.59)		
Screening result			0.665	.717
HPV-positive	42 (46.15)	39 (42.86)		
TCT abnormal	28 (30.77)	27 (29.67)		
Double positive	21 (23.08)	25 (27.47)		
Previous screening history			0.395	.530
Yes	34 (37.36)	38 (41.76)		
No	57 (62.64)	53 (58.24)		
Family history of cervical cancer			0.208	.648
Yes	12 (13.19)	14 (15.38)		
No	79 (86.81)	77 (84.62)		
Comorbidities				
Hypertension	15 (16.48)	17 (18.68)	0.156	.693
Diabetes	8 (8.79)	9 (9.89)	0.067	.796
Other gynecological diseases*	12 (13.19)	14 (15.38)	0.182	.670
Immune system diseases†	3 (3.30)	2 (2.20)	0.206	.650

* Other gynecological diseases include uterine fibroids, ovarian cysts, vaginitis, etc.

† Immune system diseases include rheumatoid arthritis, systemic lupus erythematosus, etc.

### 5.2. Comparison of follow-up completion rates and adherence levels under different nursing models

The short-term follow-up compliance rate within 3 months in the intervention group was 86.81%, significantly higher than that in the control group (64.84%), with a statistically significant difference (*P* < .001) (Table 2). Regarding the classification of compliance, the proportion of complete compliance (completed on time) in the intervention group was 71.43%, higher than that in the control group (50.55%); the noncompliance rate in the intervention group was 13.19%, lower than that in the control group (35.16%), and the difference between the groups was statistically significant (*P* = .001). These results reflect short-term diagnostic follow-up behavior after notification of abnormal screening results; sustained 6-month and 12-month follow-up compliance was not assessed in this dataset.

**Table 2:** Hierarchical comparison of patient follow-up adherence behaviors before and after nursing intervention implementation (n[%]).

Item	Control group (n = 91)	Intervention group (n = 91)	*χ*^2^ value	*P*-value
Overall compliance status			11.842	<.001
Compliant	59 (64.84)	79 (86.81)		
Noncompliant	32 (35.16)	12 (13.19)		
Classification of compliance			13.156	.001
Complete compliance (completed on time)	46 (50.55)	65 (71.43)		
Partial compliance (completed with delay)	13 (14.29)	14 (15.38)		
Noncompliance (not completed)	32 (35.16)	12 (13.19)		

### 5.3. Analysis of the constituent causes of loss to follow-up among patients who did not comply with medical advice

A total of 44 patients in this study did not complete follow-up, including 32 in the control group and 12 in the intervention group (Table 3). Analysis of reasons for loss to follow-up showed that the top 3 reasons in the control group were “forgetting” (34.38%), “fear/anxiety” (25.00%), and “being busy with work” (18.75%); the top 3 reasons in the intervention group were “being busy with work” (33.33%), “inconvenient transportation” (25.00%), and “believing follow-up unnecessary due to absence of symptoms” (16.67%). The difference in the composition of reasons for loss to follow-up between the 2 groups was statistically significant (*P* = .026). It is noteworthy that the proportions of loss to follow-up due to “forgetting” and “fear/anxiety” in the intervention group were significantly lower than those in the control group.

**Table 3:** Distribution and difference test of reasons for patient dropout between the 2 groups (n[%]).

Reason for loss to follow-up	Control group (n = 32)	Intervention group (n = 12)	*χ*^2^ value	*P*-value
Forgetting	11 (34.38)	1 (8.33)	9.284	.026
Fear/anxiety	8 (25.00)	1 (8.33)		
Busy with work	6 (18.75)	4 (33.33)		
Inconvenient transportation	3 (9.38)	3 (25.00)		
Believing follow-up unnecessary due to absence of symptoms	2 (6.25)	2 (16.67)		
Other reasons	2 (6.25)	1 (8.33)		

### 5.4. Univariate association analysis of factors influencing patient follow-up adherence behavior

To explore factors related to patient follow-up compliance, a univariate analysis was conducted with follow-up completion status as the dependent variable (Table 4). The results showed that follow-up compliance was associated with group, age, educational level, place of residence, annual income, and screening results (*P* < .05). The compliance rate in the intervention group was higher than that in the control group (*P* < .001); patients aged ≤35 years had the highest compliance proportion, while those aged ≥51 years had the lowest (*P* = .018); patients with higher educational levels, urban residence, and higher income demonstrated better compliance (*P* < .05); patients with double positive results had the highest compliance rate, while those with HPV-positive results had the lowest (*P* = .047). Previous screening history was not associated with compliance (*P* > .05).

**Table 4:** Comparison of clinical and sociodemographic characteristics between adherent and non-adherent groups (n[%]).

Factor	Compliant group (n = 138)	Noncompliant group (n = 44)	*χ*^2^ value	*P*-value
Group			11.842	<.001
Control group	59 (42.75)	32 (72.73)		
Intervention group	79 (57.25)	12 (27.27)		
Age group			8.046	.018
≤35 yr	44 (31.88)	8 (18.18)		
36–50 yr	64 (46.38)	17 (38.64)		
≥51 yr	30 (21.74)	19 (43.18)		
Educational level			9.857	.007
Junior middle school or below	47 (34.06)	26 (59.09)		
High school/technical secondary school	51 (36.96)	11 (25.00)		
Junior college or above	40 (28.99)	7 (15.91)		
Place of residence			6.324	.012
Urban	88 (63.77)	20 (45.45)		
Rural	50 (36.23)	24 (54.55)		
Annual family income			6.381	.041
<50,000 yuan	43 (31.16)	21 (47.73)		
50,000–100,000 yuan	67 (48.55)	16 (36.36)		
>100,000 yuan	28 (20.29)	7 (15.91)		
Screening result			6.124	.047
HPV-positive	55 (39.86)	26 (59.09)		
TCT abnormal	44 (31.88)	11 (25.00)		
Double positive	39 (28.26)	7 (15.91)		
Previous screening history			1.836	.175
Yes	58 (42.03)	14 (31.82)		
No	80 (57.97)	30 (68.18)		

### 5.5. Logistic regression model analysis of independent factors influencing follow-up adherence

Variables with statistical significance in the univariate analysis were included in a multivariate logistic regression model for analysis (Table 5). After adjustment for available demographic and clinical covariates, nursing intervention was independently associated with higher follow-up compliance (OR = 4.128, *P* < .001). Compliance in patients aged ≥51 years was lower than in those aged ≤35 years (OR = 0.312, *P* = .010); compliance in patients with an educational level of junior college or above was higher than in those with junior middle school education or below (OR = 3.245, *P* = .014); compliance in urban residents was better than in rural residents (OR = 2.436, *P* = .020). Annual family income and screening results showed no significant association in the multivariate analysis (*P* > .05). Given the retrospective time-grouped design, these regression findings should be interpreted as adjusted associations rather than definitive causal effects.

**Table 5:** Results of multivariate unconditional logistic regression analysis influencing follow-up adherence.

Variable	Reference group	B	S.E.	Wald *χ*^2^	*P*-value	OR value	95% CI
Group (Intervention group)	Control group	1.418	0.398	12.684	<.001	4.128	1.892–9.003
Age group				6.892	.032		
36–50 yr	≤35 yr	−0.376	0.486	0.598	.439	0.687	0.265–1.780
≥51 yr	≤35 yr	−1.164	0.454	6.577	.010	0.312	0.128–0.759
Educational level				7.214	.027		
High school/technical secondary school	Junior middle school or below	0.892	0.468	3.632	.057	2.440	0.975–6.108
Junior college or above	Junior middle school or below	1.177	0.480	6.018	.014	3.245	1.267–8.312
Place of residence (Urban)	Rural	0.890	0.384	5.376	.020	2.436	1.148–5.171
Annual family income				2.891	.236		
50,000–100,000 yuan	<50,000 yuan	0.512	0.412	1.544	.214	1.669	0.744–3.743
> 100,000 yuan	<50,000 yuan	0.694	0.547	1.611	.204	2.002	0.686–5.845
Screening result				3.784	.151		
TCT abnormal	HPV-positive	0.487	0.446	1.192	.275	1.627	0.679–3.900
Double positive	HPV-positive	0.894	0.535	2.791	.095	2.445	0.857–6.978
Constant		−0.845	0.628	1.812	.178	0.430	

### 5.6. Heterogeneity analysis of the effect of nursing intervention across different subgroups

Subgroup analysis further evaluated the effect of nursing intervention in different populations (Table 6). The analysis showed that nursing intervention effectively improved follow-up compliance rates across all populations. In different age groups (≤45 years and >45 years), the compliance rates in the intervention group were significantly higher than those in the control group (*P* < .05). Regarding screening results, the intervention effect was significant in the HPV-positive population (*P* = .003), while the difference between groups in the TCT abnormal/double positive population did not reach statistical significance (*P* = .056). Among both urban and rural populations, the compliance rates in the intervention group were significantly higher (*P* < .05), with a greater magnitude of improvement observed in the rural population.

**Table 6:** Subgroup analysis of nursing intervention effectiveness stratified by age, screening result, and place of residence.

Subgroup	Control group compliance rate	Intervention group compliancerate	*χ*^2^ value	*P*-value
Age				
≤45 yr (n = 98)	68.42% (39/57)	87.80% (36/41)	5.038	.025
>45 yr (n = 84)	58.82% (20/34)	86.00% (43/50)	8.141	.004
Screening result				
HPV-positive (n = 81)	54.76% (23/42)	84.62% (33/39)	8.513	.003
TCT abnormal/double positive (n = 101)	73.47% (36/49)	88.46% (46/52)	3.662	.056
Place of residence				
Urban (n = 108)	73.08% (38/52)	91.07% (51/56)	5.982	.014
Rural (n = 74)	51.28% (20/39)	79.41% (27/34)	6.944	.008

## 6. Discussion

This study used a retrospective cohort design based on time grouping to evaluate the impact of nursing intervention on follow-up compliance among patients with abnormal cervical cancer screening results. The results showed that the short-term follow-up compliance rate in patients receiving systematic nursing intervention was 86.81%, significantly higher than the 64.84% in the routine nursing group. Furthermore, the intervention group had a higher proportion of complete compliance and a lower proportion of noncompliance, suggesting that nursing intervention was associated with improved follow-up compliance.^[12]^ Multivariate analysis further showed that nursing intervention was independently associated with improved compliance; the likelihood of follow-up compliance in the intervention group was 4.128 times that of the control group after adjustment for available covariates. However, because grouping was based on the pre-implementation and post-implementation periods rather than randomization, the observed difference should be interpreted as an association and not as proof of a purely causal effect. This finding is consistent with results from related domestic and international studies. Previous research implementing continuous nursing care for patients with abnormal cervical cancer screening results showed that the follow-up compliance rate in the intervention group was significantly higher than that in the control group; other studies using telephone counseling interventions also significantly improved follow-up compliance in low-income populations.^[13]^ Building on this, the present study provides practical evidence for integrating systematic nursing management into early diagnosis and treatment workflows for cervical cancer, while also recognizing the methodological limitations of a time-grouped retrospective design.^[14]^

Analyzing the mechanism of action, the nursing intervention measures in this study were multidimensional and systematic, forming a closed-loop management system that included establishing follow-up records, intensifying health education, proactively reminding about follow-up, providing continuous psychological support, and offering green channel services.^[15]^ Firstly, establishing electronic follow-up records made patient information traceable and trackable, laying the foundation for subsequent proactive intervention. Secondly, one-on-one intensive health education corrected common patient cognitive misconceptions, particularly by emphasizing that “precancerous lesion does not equal cancer” and the “curability of early diagnosis and treatment,” effectively alleviating patient fear.^[16]^ Analysis of reasons for loss to follow-up revealed that the proportion of loss to follow-up due to “fear/anxiety” in the control group was as high as 25.00%, compared to only 8.33% in the intervention group, indicating that health education played a significant role in alleviating patients’ psychological burden. Thirdly, the 1-week advance telephone or SMS reminder mechanism directly targeted the primary reason for loss to follow-up in the control group, “forgetting” (accounting for 34.38%); after the intervention, the proportion of loss to follow-up due to forgetting dropped to 8.33%, demonstrating that proactive reminders are an effective means of improving compliance.^[17]^ Additionally, the overdue follow-up mechanism provided a second chance for patients who missed appointments due to objective reasons, reducing loss to follow-up caused by factors such as being busy with work or inconvenient transportation. Finally, the green channel service shortened patient waiting times and reduced the risk of dropout associated with cumbersome consultation procedures.^[18]^

From an international perspective, improving follow-up after abnormal cervical cancer screening is closely aligned with the World Health Organization strategy for cervical cancer elimination, which emphasizes not only vaccination and screening coverage but also timely treatment for women with cervical disease. In organized screening systems in many high-income countries, recall and follow-up are often supported by centralized registries, invitation systems, and relatively stable referral pathways. In many low- and middle-income or resource-constrained settings, however, loss to follow-up is more often related to fragmented referral processes, out-of-pocket costs, long travel distance, and limited health literacy. International studies of telephone counseling, SMS reminders, lay health worker involvement, and patient navigation have shown that active outreach can improve screening or diagnostic follow-up, especially in underserved groups.^[19–21]^ The findings of the present study are generally consistent with this international evidence, but the intervention was delivered within a municipal maternal and child health hospital in China. Therefore, the transferability of the findings may be strongest in similar hospital-based screening and follow-up systems; settings with different financing mechanisms, community health networks, or registry infrastructure may require adaptation before implementation.

The time-grouped design is a practical feature of real-world implementation research, but it may introduce time bias. Potential secular changes during the study period include increased public awareness of HPV and cervical cancer, changes in clinic workload, improved experience of medical staff, greater appointment availability, or adjustments in institutional follow-up pathways. Although the baseline characteristics of the 2 groups were comparable and multivariate adjustment was performed, unmeasured time-period differences may still have contributed to the observed improvement. Therefore, the manuscript avoids attributing all differences solely to nursing intervention. Future prospective studies using concurrent controls, cluster randomization, or interrupted time-series methods could more effectively separate intervention effects from secular trends.

Short-term and long-term compliance should also be distinguished. The present study assessed completion of colposcopy or treatment within 3 months after notification, which reflects immediate diagnostic follow-up and is highly relevant to the prevention of delayed diagnosis. Long-term compliance at 6 or 12 months would reflect sustained surveillance, repeated testing, treatment completion, and management of recurrent barriers. Reminder-based and education-based interventions may have strong short-term effects on forgetting and anxiety, but their long-term effects may be weakened by work constraints, transportation costs, reduced contact intensity, and the absence of symptoms. Therefore, the current results should not be extrapolated automatically to long-term follow-up compliance, and future studies should include 6-month and 12-month outcomes.

Analysis of the reasons for loss to follow-up showed a significant difference in the composition of these reasons between the 2 groups. The control group was primarily characterized by forgetting and fear/anxiety, which are subjective factors amenable to improvement through nursing intervention. In contrast, the intervention group was mainly characterized by being busy with work and inconvenient transportation, which are objective social factors. This shift suggests that nursing intervention effectively addresses patients’ subjective barriers, but objective factors such as work schedules and transportation issues still need to be tackled through systemic measures like optimizing healthcare service models and advancing tiered medical diagnosis and treatment.^[19]^ It is noteworthy that 16.67% of patients in the intervention group were still lost to follow-up due to “believing follow-up unnecessary due to absence of symptoms,” indicating that some patients still hold misconceptions about the disease, and the depth and continuity of health education require further strengthening.

Subgroup analysis further revealed differential effects of nursing intervention across different populations. In the HPV-positive population, the compliance rate in the intervention group increased by nearly 30 percentage points compared to the control group, demonstrating a highly significant effect, whereas the magnitude of improvement was relatively smaller in the TCT abnormal/double positive population. This may be because the condition is relatively more explicit in patients with double positive results, and the level of attention from both doctors and patients is inherently higher; consequently, the compliance rate under routine nursing care has already reached a relatively high level, limiting the incremental effect of the intervention. In contrast, patients with HPV-positive results, often lacking symptoms and having insufficient awareness of the harm of viral infection, tend to neglect follow-up easily, making the “awakening effect” of nursing intervention more pronounced. This finding suggests that the HPV-positive population should be a key focus group for nursing intervention. Subgroup analysis by place of residence showed that the improvement due to nursing intervention was greater in the rural population than in the urban population, indicating that nursing intervention helps bridge the urban-rural health disparity and holds significant public health importance.^[21]^

This study has several limitations. First, as a single-center retrospective cohort study based on time grouping, patients in the control and intervention groups were enrolled in different calendar periods. Residual time bias and unmeasured secular changes cannot be excluded, even though baseline characteristics were balanced and multivariate adjustment was performed. Second, selection bias may exist because the sample came from 1 municipal maternal and child health hospital and excluded patients with missing key information or invalid contact details; therefore, the findings may not represent women screened in community clinics, private hospitals, rural outreach settings, or institutions without similar nursing resources. Third, information bias may arise from inaccuracies or incomplete documentation in retrospective HIS, laboratory, pathology, and follow-up records. Fourth, reasons for loss to follow-up relied partly on telephone re-inquiry when contemporaneous records were incomplete; therefore, recall bias or social desirability bias may have affected patient-reported reasons. Fifth, the endpoint was short-term follow-up compliance within 3 months; long-term compliance at 6 or 12 months was not evaluated, so the persistence of the intervention effect remains unclear. Sixth, the nursing intervention was a comprehensive package comprising multiple components, including record establishment, health education, follow-up reminders, psychological support, and green channel services. This study could not conduct a split or component analysis, making it impossible to determine which intervention component was most critical or whether some components had synergistic effects. Finally, the applicability of the results should be limited to similar medical environments with hospital-based screening, electronic records, specialized nurses, and telephone/SMS reminder resources. International settings or lower-resource systems may require adaptation according to local referral pathways, financing mechanisms, and patient navigation capacity. Future multicenter prospective studies should evaluate 6-month and 12-month compliance, compare individual intervention components, and assess cost-effectiveness.

In conclusion, this study suggests that systematic nursing intervention is associated with significantly higher short-term follow-up compliance among patients with abnormal cervical cancer screening results and may reduce loss to follow-up due to forgetting and fear. The intervention appeared particularly beneficial in HPV-positive and rural populations. Age, educational level, and place of residence were also important factors associated with follow-up compliance. In the early diagnosis and treatment of cervical cancer, systematic nursing management may be considered as an important component of routine workflows in similar medical environments, with differentiated intervention strategies for older patients, patients with lower educational levels, and rural residents. However, because the study was retrospective, time-grouped, single-center, and limited to a 3-month outcome window, the findings should be interpreted cautiously. Future multicenter prospective studies with concurrent controls, longer follow-up periods, component analysis, and cost-effectiveness evaluation are needed to provide stronger evidence for optimizing nursing resources and follow-up management.

## Author contributions

**Conceptualization:** Xiaohui Deng, Xiaoqing Pang, Zhengyan Dai.

**Data curation:** Xiaohui Deng, Xiaoqing Pang, Zhengyan Dai.

**Formal analysis:** Xiaohui Deng, Xiaoqing Pang, Zhengyan Dai.

**Funding acquisition:** Zhengyan Dai.

**Investigation:** Zhengyan Dai.

**Writing – original draft:** Zhengyan Dai.

**Writing – review & editing:** Zhengyan Dai.
